# Clinical and epidemiological features of high-risk human papillomavirus infection in patients with cervical intraepithelial lesions

**DOI:** 10.1186/s12905-023-02583-x

**Published:** 2023-09-01

**Authors:** Yu-Qin Ding, Jie Yu, Run-Qiu Wang, Lin Sang

**Affiliations:** Department of obstetrics and gynecology, The Second People’s Hospital of Hefei, Hefei Hospital Affiliated to Anhui Medical University, Guangde Road, Yaohai District, 230011 Hefei, China

**Keywords:** Age, High-grade squamous intraepithelial lesions (HSIL), Human papillomavirus, Low grade squamous intraepithelial lesions (LSIL)

## Abstract

**Objective:**

In this study, we analyzed the clinical and epidemiological features of high-risk human papillomavirus (HR-HPV) infection in patients with cervical intraepithelial lesions.

**Methods:**

Retrospective analysis was performed on the clinical data of 240 cases of histologically confirmed cervical squamous intraepithelial lesions to determine any correlation between HPV infection characteristics, age distribution, and cervical epithelial lesions.

**Results:**

Patients between the ages of 31 and 40 with cervical intraepithelial lesions were more likely to have high-grade squamous intraepithelial lesions (HSIL; 40.7%) than low-grade squamous intraepithelial lesions (LSIL; 31.3%) (*P* < 0.05). In patients with HSIL, HR-HPV16, HR-HPV33, and HR-HPV52 were the most common types of HPV infection, while in patients with LSIL, HR-HPV16, HR-HPV52, and HR-HPV58 were the most common types of HPV infection. The highest percentage of single infections occurred in the HSIL group (69.6%), followed by the LSIL group (68.8%). HSIL was present in a significant number of patients (28.6%) aged 30 years and above who tested positive for 12 HPV types but negative for TCT.

**Conclusion:**

The prevalence of HSIL is greatest in younger patients. Patients with cervical epithelial lesions typically have a single infection of a high-risk HPV genotype—HR-HPV16, HR-HPV33, HR-HPV52, or HR-HPV58. Patients aged 30 years and above who test positive for one of 12 types of HPV but negative for TCT are at increased risk for developing HSIL. In order to detect cervical lesions early and begin treatment without delay, colposcopy should be performed regardless of whether or not a high-risk HPV infection is present.

## Introduction

Cervical cancer is by far the most prevalent human papillomavirus (HPV)-related disease, and more than 90% of cervical cancer cases can be attributed to infection with high-risk HPV [1]. Studies show that roughly 20% of the population carries high-risk HPV. The three most prevalent HPV genotypes are HR-HPV52, HR-HPV16, and HR-HPV58 [2, 3]. The most common type of HPV infection in China is HR-HPV52, and it is found primarily in the eastern, central, southern, and southwestern regions of the country [3] A persistent infection with high-risk HPV is linked to both the development and spread of cervical cancer. HPV is linked to high grade squamous intraepithelial lesion (HSIL), an abnormality of squamous cells. These include CIN2, CIN3, moderate and severe dysplasia, and carcinoma in situ, all of which were previously used terms [4]. Regardless of the results of HPV co-testing, immediate excisional procedure or colposcopy is recommended for women over the age of 24 who have a positive HSIL pap test result and no extenuating circumstances. A diagnostic excisional procedure is recommended if the colposcopic examination is insufficient. Ablation of the transformation zone or excision is considered acceptable if HSIL (CIN2, CIN3, or CIN2-3) is confirmed on biopsy and an adequate colposcopy was performed. It is not recommended that pregnant women who test positive for HSIL have excisional treatment; only colposcopy is safe. A literature review concluded that birth defects, low birth weight, and premature membrane rupture before 37 weeks of pregnancy were more common in women who had CIN surgery compared to those who did not [5]. There is widespread agreement that treating high-risk HPV strains early can avert the progression of cervical cancer and improve its treatment. Cervical cancer screening tools, such as high-risk HPV typing and quantitative detection and thinprep cytology test (TCT), have improved and standardized in recent years.

In this study, we aimed to determine the association between HPV infection characteristics, age, and cervical intraepithelial lesions, as well as the association between the high-risk HPV positive population with negative cytology results and cervical intraepithelial lesions, using a retrospective analysis of clinical data from 240 patients with cervical intraepithelial lesions.

## Patients and methods

### Data and methods

Clinical data: The clinical data of 240 patients with cervical intraepithelial lesions diagnosed with cervical cancer using the “three-step” screening method in the gynecological clinic of the Second People’s Hospital of Hefei were collected from May 2021 to August 2022. The patients were then divided into two groups—high-grade squamous intraepithelial lesion (HSIL; 115 patients) group and low-grade squamous intraepithelial lesion (LSIL; 125 patients) group.

Inclusion criteria: (1) Age 18 years and above; (2) Complete clinical data, including gravidity, parity, TCT, HPV test results, and pathological results of colposcopic positioning biopsy, are available.

Exclusion criteria: Patients with incomplete clinical data; Patients with a history of treatment for cervical precancerous lesions and hysterectomy due to cervical precancerous lesions; Patients with vaginal intraepithelial lesions; Acute genital tract inflammation; Pregnant women and women clinically suspected of cancer.

None of the patients had been vaccinated against HPV. All patients had signed an informed consent form prior to the colposcopy and cervical biopsy.

### Examination methods

#### HPV and TCT testing

For at least three days prior to sample collection, patients were instructed to abstain from any sex or vaginal drug use. A vaginal speculum was used to expose the cervix while the patient was in the lithotomy position. The vaginal secretions and external cervical orifice were cleaned with long cotton swabs. The cervical orifice was brushed with special cervical brushes designed to detect HPV and TCT, and then the brushes were placed in sterile bottles for later use.

HPV was genotyped using a human papillomavirus genotyping assay kit (PCR-reverse dot blot assay), identifying high-risk HPV types—16, 18, 31, 33, 35, 39, 45, 51, 52, 56, 58, 59, 66, and 68; as well as low-risk HPV types—6 and 11.

TCT was utilized for cytological analysis. The cytological diagnosis was made using the 2001 cervical cytology classification system (TBS classification system), which included NILM, ASC-US, ASC-H, LSIL, HSIL, AGC, SCC, and adenocarcinoma.

1.2.2 Colposcopy and cervical biopsy: The Leisegang photoelectric colposcope (Germany) was used. Specialists in colposcopy with more than a year of experience in the field performed all procedures according to standard protocol and reported their findings using colposcopy terminology established in 2011 by the International Federation of Cervical Pathology and Colposcopy (IFCPC). The suspected cervical lesion was biopsied using biopsy forceps and colposcopic positioning. The highest-grade pathological result from the cervical biopsy was used as the final pathological diagnosis, which was determined using the 2014 (4th edition) WHO classification criteria for female reproductive organ tumors. LSIL included CINI and immunohistochemically P16-negative CINII, whereas HSIL included CINII and CINIII that were immunohistochemically P16-positive.

### Statistical method

The data from the measurements are expressed as mean ± standard deviation (‾x± s). The independent sample *t*-test was used to compare the groups, the counting data are expressed as (n, %), and the chi-squared test was conducted. The differences were statistically significant if *P* < 0.05.

## Results

### General data comparison between the HSIL and LSIL groups

The 240 patients enrolled had cervical intraepithelial lesions; 115 had HSIL and were on average 37.38±9.63 years old, while 125 patients had LSIL and were on average 39.06±11.60 years old. Age was significantly different between the two groups (*P* < 0.05), whereas pregnancy and parity were not significantly different (*P* > 0.05). Further grouping analysis showed the peak age of HSIL between 31 and 50 years, and the incidence of HSIL in patients aged between 31 and 40 years was significantly higher than that of LSIL (*P* < 0.05), as shown in Table 1. However, the incidence of HSIL in patients aged 41 and older was lower than that of LSIL, but the difference was not statistically significant (*P* < 0.05), as shown in Table 1.

**Table 1:** Comparison of general data between the two groups of patients

Groups	Cases	HR-HPV infection%	Age (years)	Age group (years)				Gravidity	Parity
				≤ 30	31–40	41–50	> 50		
LSIL-CIN 1 group	125	92.0	39.06±11.60	19(16.5)	32(31.3)	34(29.6)	30(26.1)	2.82±1.77	1.54±1.03
HSIL-CIN 2, CIN 3 group	115	98.3	37.38±9.63	17(15.0)	46(40.7)	26(23.0)	24(21.0)	2.72±1.50	1.51±0.84
*P*			0.009	0.760	0.040	0.230	0.389	0.068	0.156

Note: LSIL: Low-grade cervical intraepithelial lesion; HR-HPV : high-risk human papillomavirus; HSIL: High-grade cervical intraepithelial lesion;CIN: cervical intra-epithelial neoplasia

### Comparing the types of high-risk HPV infections between the HSIL and LSIL groups

In patients with HSIL, the most common types of HPV infection were HR-HPV16, HR-HPV33, and HR-HPV52, accounting for 45.5%, 12.8%, and 10.9%, respectively; in patients with LSIL, the most common types of HPV infection were HR-HPV16, HR-HPV52, and HR-HPV58, accounting for 25.3%, 20.7%, and 14.0%, respectively. The infection rates of HR-HPV16 and HR-HPV33 were significantly higher in the HSIL group compared to the LSIL group, and the differences were statistically significant (*P* < 0.05) (Table 2).

**Table 2:** Comparison of single high-risk HPV infection type between the two groups (n, %)

HR-HPV infection type	LSIL-CIN 1 group(n = 125)	HSIL-CIN 2, CIN 3 group(n = 115)	X^2^	*P*
HR-HPV16	38(25.3)	71(45.5)	13.579	0.000
h-HPV18	13(8.7)	9(5.8)	0.962	0.327
h-HPV31	4(2.7)	5(3.2)	0.078	0.780
h-HPV33	5(3.3)	20(12.8)	9.174	0.002
h-HPV35	6(4.0)	4(2.6)	0.284	0.594
h-HPV39	4(2.7)	3(1.9)	0.189	0.664
h-HPV45	2(1.3)	0(0.0)	0.544	0.461
h-HPV51	7(4.7)	7(4.5)	0.006	0.940
h-HPV52	31(20.7)	17(10.9)	5.518	0.019
h-HPV56	7(4.7)	4(2.6)	0.708	0.400
h-HPV58	21(14.0)	10(6.4)	4.838	0.028
h-HPV59	4(2.7)	2(1.3)	0.157	0.692
h-HPV66	1(0.6)	0(0.0)	0.000	0.989
h-HPV68	7(4.7)	4(2.6)	0.523	0.469
Total	150(100)	156(100)		

Note: LSIL: Low-grade cervical intraepithelial lesion; HR-HPV : high-risk human papillomavirus; HSIL: High-grade cervical intraepithelial lesion; CIN: cervical intra-epithelial neoplasia

### Comparison between single and multiple HPV infections and cervical intraepithelial neoplasia

Multiple infections accounted for 27.2% of patients infected with HPV, while single infections comprised the largest proportion in both the HSIL and LSIL groups. The incidence of double and triple infections was greater among the HSIL group than among the LSIL group, but the difference was not statistically significant (*P* > 0.05). The LSIL group had a higher proportion of patients who were HPV-negative than the HSIL group, and the difference was statistically significant (*P* < 0.05) (Table 3).

**Table 3:** Relationship between HPV single/multiple infection and cervical intraepithelial lesions (n, %)

HR-HPV infection type	LSIL-CIN 1 group(n = 125)	HSIL-CIN 2, CIN 3 group(n = 115)	X^2^	*P*
HPV-	10(8.0)	2(1.7)	4.943	0.026
h-HPV single infection	86(68.8)	80(69.6)	0.016	0.898
h-HPV double infection	23(18.4)	23(20.0)	0.099	0.753
h-HPV triple infection	6(4.8)	10(8.7)	2.147	0.143

Note: LSIL: Low-grade cervical intraepithelial lesion; HR-HPV : high-risk human papillomavirus; HSIL: High-grade cervical intraepithelial lesion; CIN: cervical intra-epithelial neoplasia

### Comparison of TCT-negative cervical intraepithelial lesions and different high-risk HPV types in patients over 30 years old

In addition, we analyzed the characteristics of high-risk HPV infection in patients over the age of 30 with negative TCT results. The results revealed that the HR-HPV16 infection group had the highest HSIL detection rate. The proportion of patients with HSIL in the other 12 types of infection group accounted for 28.6% (Table 4).

**Table 4:** Comparison of cervical intraepithelial lesions with TCT negative and different high-risk HPV types in patients over 30 years old

HR-HPV infection type	Cases	HSIL-CIN 2, CIN 3	LSIL-CIN 1
h-HPV16 infection^*^	95	65(68.4)	30(31.5)
HR-HPV18 infection^*^	17	7(41.2)	10(58.8)
Other 12 types of infections	70	20(28.6)	50(71.4)

Note: *: Indicates that other 12 types of infections can be included.; TCT: thin-prep cytologic tests;

LSIL: Low-grade cervical intraepithelial lesion; HR-HPV: high-risk human papillomavirus; HSIL: High-grade cervical intraepithelial lesion; CIN: cervical intra-epithelial neoplasia

## Discussion

The results of this study showed that HSIL was more prevalent than LSIL among patients diagnosed with cervical intraepithelial lesions. HR-HPV16, HR-HPV33, and HR-HPV52 were the most frequent type of HPV infection in patients with HSIL, while HR-HPV16, HR-HPV52, and HR-HPV58 were the most common types of HPV infection in patients with LSIL. The highest percentage of single infections occurred in the HSIL group, followed by the LSIL group. HSIL was present in a significant number of patients (28.6%) aged 30 years and above who tested positive for 12 HPV types but negative for TCT.

In a multicenter study in New Mexico, HR-HPV16, HR-HPV33, and HR-HPV31 had the highest positive predictive value for CIN2+, followed by HR-HPV18, HR-HPV35, and HR-HPV58 [6]. The most prevalent HPV types in CINI in 2020, according to a survey, were HR-HPV52 (20.31%), HR-HPV16 (16.81%), HR-HPV58 (14.44%), HR-HPV18 (6.44%), and HR-HPV53 (5.76%). HR-HPV16 infection rate was the highest (45.69%), followed by HR-HPV58 (15.50%), HR-HPV52 (11.74%), HR-HPV33 (9.35%), and HR-HPV31 (4.34%) [7], suggesting that HR-HPV16 is the most prevalent oncogenic genotype, and the distribution of HPV genotypes varies by region. This indicates that HPV vaccine administration should be adapted to regional needs. The results of this study revealed that among women in the LSIL group, 92% were infected with high-risk HPV, the most common strains of which were HR-HPV16, HR-HPV52, and HR-HPV58. In the HSIL group, 98.3% were infected with high-risk HPV, with HR-HPV16 being the most common type of HPV present, followed by HR-HPV33 and HR-HPV52. As a result, the prevalence of various HPV genotypes in cervical lesions of varying severities, as well as the predominant types of HPV infection in the LSIL and HSIL groups, are clearly distinguishable. When a patient has a normal cytology result but is positive for HR-HPV33, HR-HPV52, or HR-HPV58, a colposcopy examination and positioning biopsy of suspicious lesions under colposcopy should be performed to rule out cervical HSIL.

The prevalence of HPV infection is thought to be highest in young and middle-aged women. Young women, especially those between the ages of 25 and 35, may be more likely to contract HPV if they engage in more frequent sexual activity [8]. Women between the ages of 55 and 65 have a significantly higher risk of contracting HPV due to low levels of education and weakened cervical resistance caused by autoimmune disease and hormones [9]. However, the detection rate of HSIL varies among patients of different ages. There are clear differences in the age distribution of patients with various grades of cervical lesions, with the highest detection rate of HSIL occurring in patients under the age of 40 who only have a single HPV infection [10], while another study demonstrated that the detection rate of HSIL is highest in individuals aged 41 to 50 years (32.37%) [11]. Yang et al. compared the HSIL detection rates among individuals aged between 35 and 45, 46 to 55, and 56 to 65 [9]. The rate of CINII + detection increased with age, but the difference was not statistically significant (*P* = 0.414). Based on our findings, the average age of the HSIL group was 37.38±9.63 years old, which is significantly lower than the average age of the LSIL group, which was 39.06±11.60 years old. Among patients aged between 31 and 40 years, the incidence of HSIL was 40.7%, which is significantly higher than that of the LSIL group (31.3%), possibly due to the intraepithelial cervical lesions being more prevalent among the younger age group. The relatively high education levels of young women, combined with the successful promotion and popularization of cervical screening, have led to a greater public awareness of cervical precancerous lesions. Many high-grade squamous lesions have been detected and effectively treated, and few patients have contracted disease as a direct result of skipping cervical screening. Therefore, the incidence of HSIL is lower in patients older than 40 than that of LSIL.

According to research, over 80% of sexually active people have been infected with HPV at some point [12], and persistent infection with high-risk HPV is strongly associated with intraepithelial lesions in the cervical epithelium. However, there is no conclusive evidence that having multiple HPV infections raises the risk of developing precancerous cervical lesions. Patients with cervical epithelial lesion typically have a single high-risk HPV infection, and these infections are most often HR-HPV16, HR-HPV33, HR-HPV52, or HPV58 [13]. Xiang et al. found that the rates of single, double, and multiple HPV infections were 8%, 1%, and 0% among CINI patients, 24%, 7%, and 1% among CINII-III patients, and 57%, 25%, and 3% among cervical cancer patients, respectively [14]. We hypothesize that the proportion of single infections, double infections, and multiple infections of HPV would increase with the severity of cervical lesions; however, no statistically significant difference was found, which may be due to the small sample size. Another study found that multiple high-risk HPV infection rates were highest in cervical CINIII lesions [15], indicating that multiple HPV types appear to play a synergistic role in the development of cervical cancer [16]. Maria et al. examined the results of 900 patients who underwent cervical cytology, HPV typing test, and colposcopy biopsy. The results indicated that the lesion type with the highest percentage of high-risk HPV single infection was SCC (100%), followed by CIN3 (78%), and the lesion types with the highest percentages of multiple infections were CIN1 (60.4%), CIN2 (43.7%), and CIN3 (22.1%). It is assumed that a single HPV infection poses a greater risk of developing into SCC than multiple infections [17]. Other studies also show that multiple high-risk HPV infections are not a direct risk factor for the development of cervical cancer [18, 19], as the mechanism may involve inter-gene competition, or an enhanced immune response triggered by multiple infections. Multiple HPV infections did not significantly increase the risk of cervical intraepithelial lesions (*P* > 0.05).

In 2018, the United States Preventive Services Task Force (USPSTF) recommended combining TCT and HPV testing in cervical cancer screening for individuals aged between 30 and 65 [20]. Therefore, women over the age of 30 make up the bulk of the population screened with the combined approach; however, the cytological screening is affected by a number of variables, such as the sampling standard of the specimen, the retention time, and the subjective judgement of the pathologist. In high-risk HPV-positive individuals with a negative TCT, the missed diagnosis rate of intraepithelial cervical lesions is high because of the test’s low sensitivity. HSIL is also more prevalent among patients older than 30 who test positive for one of 12 types of HPV but are negative for TCT [13]. According to studies, the risk of CIN2 + in TCT-negative, HR-HPV16-positive women is 13.6%, while the risk of CIN2 + in TCT-negative, HR-HPV18-positive women is 7% [21]. A five-year study by Uijterwaal et al. found that 11.4% of non-HR-HPV 16/18-positive women with normal cytology are also at risk for HSIL [22]. Multivariate analyses in other studies demonstrate that the risk of high-grade squamous lesions with negative cytology but persistent high-risk HR-HPV31 and 33 positivity is 1.53 times and 2.02 times higher, respectively, and the difference is statistically significant [23]. The results of our study revealed that the detection rate of HSIL was higher in HR-HPV16 and HR-HPV18 infection than in the other 12 types of HPV infection; however, the detection rate of HSIL in TCT-negative and the other 12 types of HPV-positive was only 28.6%, indicating that simple cytological screening may miss the diagnosis of some HSIL lesions.

When a cervical biopsy reveals HSIL-CINII or CINIII, patients receive additional treatment with LEEP. However, for patients with early stage cervical cancer, radical hysterectomy and plus pelvic node dissection are the main methods of treatment [24]. For low-risk patients, there is mounting evidence that laparoscopic radical hysterectomy has equivalent 10-year outcomes to open surgery [25]. This has profound implications for patient care, underscoring the need for future research to examine whether treatment success is linked to HPV infection.

Limitations: In this study we aimed to determine the most common types of HR-HPV infection among patients diagnosed with cervical intraepithelial lesions. For young patients aged 30 and higher, colposcopy should be performed for early detection of cervical lesions and timely treatment regardless of high-risk HPV infection status. Nonetheless, the study suffered from a lack of statistical power due to the small sample size and a single-center design. Therefore, additional confirmation with a larger sample size in multiple centers is recommended.

In conclusion, the incidence of cervical lesions varies with age, and HPV infections other than HPV16/18, should also be considered, especially when cytological screening is negative. Due to the fact that the risk of developing cervical intraepithelial lesions is not greatly increased by a second or subsequent infection with a high-risk HPV strain, those at high risk should be closely monitored, subjected to regular screenings, and administered an HPV vaccine at the optimal time to prevent cervical cancer.

## Data Availability

The data that support the findings of this study are available from the corresponding author upon reasonable request.

## Abbreviations

HR-HPV: high-risk human papillomavirus
HSIL: High-grade cervical intraepithelial lesion
LSIL: Low-grade cervical intraepithelial lesion
TCT: Thinprep cytologic test
PCR: Polymerase Chain Reaction
NILM: Negative for intraepithelial lesion or malignancy
ASC-US: Atypical squamous cells of undetermined significance
ASC-H: Atypical squamous cells cannot exclude high-grade squamous intraepithelial lesion
AGC: Atypical glandular cells
SCC: squamous cell carcinoma
CIN: cervical intra-epithelial neoplasia
USPSTF: US Preventive Services Task Force
